# Dynamical Landscape of Heart Rhythm in Long-Term Heart Transplant Recipients: A Way to Discern Erratic Rhythms

**DOI:** 10.3389/fphys.2018.00274

**Published:** 2018-04-09

**Authors:** Joanna Wdowczyk, Danuta Makowiec, Marcin Gruchała, Dorota Wejer, Zbigniew R. Struzik

**Affiliations:** ^1^First Cardiology Clinic, Medical University of Gdańsk, Gdańsk, Poland; ^2^Faculty of Mathematics, Physics and Informatics, Institute of Theoretical Physics and Astrophysics, University of Gdańsk, Gdańsk, Poland; ^3^Faculty of Mathematics, Physics and Informatics, Institute of Experimental Physics, University of Gdańsk, Gdańsk, Poland; ^4^Laboratory for Advanced Brain Signal Processing, RIKEN Brain Science Institute, Wako, Japan; ^5^Graduate School of Education, University of Tokyo, Tokyo, Japan

**Keywords:** heart rate variability, entropic measures, heart transplant patients, erratic rhythm, heart rate fragmentation, autonomic nervous system

## Abstract

It is commonly believed that higher values of heart rate variability (HRV) indices account for better organization of the network of feedback reflexes driving an organism's response to actual bodily needs. In order to evaluate this organization in heart transplant (HTX) recipients, 58 nocturnal Holter signals of 14 HTX patients were analyzed. Their dynamical properties were evaluated by short-term HRV indices and measures grounded on entropy. Estimates grouped according to the patients' clinical progress: free of complications versus with complications, and arranged in order of the length of time since the HTX, lead us to the conclusion that higher HRV is associated with a worse outcome for HTX patients. Moreover, short-term HRV indices that are constant, rather than increasing over time, serve well in the prognosis of the future state of a HTX patient. These findings suggest that increases observed in HRV indices are related to erratic rhythms resulting from remodeling of the cardiac tissue (including heterogeneous innervation) in long-term HTX patients. Therefore, we hypothesize that dynamical landscape markers (entropy and fragmentation measures together with the short-term HRV indices) can serve as a tool in the exploration of the genesis of (non-respiratory sinus) arrhythmia.

## 1. Introduction

Heart transplant (HTX) recipient heart rhythm is specific due to the complete denervation of the donor heart after surgery. The basic source of heart rate variability (HRV) in a healthy heart, the autonomic nervous system (ANS), is cut off from direct influence on the heart. The heart displays an intrinsic rhythm of around 110 beats/min, which is determined by spontaneous depolarization of pacemaker cells in the sinoatrial node (SAN). Accordingly, early post-HTX heart rate (HR) at rest is higher than normal. However, the ANS regulation is served indirectly via, for example, circulating hormones. With the passing of time after the surgery, the heart rhythm of the HTX patient changes. As reinnervation of the recipient occurs over time post-HTX, the intrinsic heart rhythm has less influence on the resting HR, reducing the HR to a lower value and leading to partially increased HRV values (Awad et al., 2016). Reinnervation of the heart is clinically important, resulting in improved exertional HR response, improved contractile function, and more sufficient myocardial blood flow, providing better quality of life (Grupper et al., 2017). Therefore, for years it was believed that an increase in HRV indicated the proper process of donor heart adaptation to a new organism (Grupper et al., 2017). However, this is not entirely clear (Nicolini et al., 2012). After heart transplantation, the allograft undergoes characteristic alterations in myocardial structure, including hypertrophy, increased ventricular stiffness, ischemia, and inflammation which, together with the natural process of aging, may lead to vasculopathy and fibrosis of the donor heart (Alraies and Eckman, 2014; Coelho-Filho et al., 2016). The scale and intensity of the spontaneous process of reinnervation in the myocardial tissue progresses irregularly, and differs greatly from patient to patient (Radaelli et al., 1996; Bengel et al., 2004; Viola et al., 2004; Cornelissen et al., 2012; Vanderlaan et al., 2012; Awad et al., 2016; Grupper et al., 2017).

Additionally, with the passing of time after the surgery, it becomes very likely that the suture lines, which initially isolate the native heart parts from the donor heart, lose their role. All together, this impacts on progressive alternations in the myocardial structure of the donor heart, which influences the propagation of activation wavefronts. Therefore, a variety of arrhythmias—abnormal heart rhythms, may occur (Thajudeen et al., 2012; Hamon et al., 2014). As all the HTX patients in our clinic are under permanent clinical control, their electrocardiographic signals and relevant clinical and laboratory data are collected during scheduled routine follow-up visits. In consequence, our clinic possesses numerous signals recorded from the same patient. This means that we can observe the follow-up path and the evolution of the heart rhythm of each patient separately. We have presented examples of such investigations in our previous papers; see Wdowczyk et al. (2016) and Makowiec et al. (2016). It seems that a group of HTX patients can be regarded as a perfect sample for observations of the birth of erratic rhythms and their further development into full arrhythmia.

It has been proposed that changes in RR-intervals may encode the short-term dependence of heartbeat dynamics (Makowiec et al., 2015b; Costa et al., 2017). Accordingly, statistics based on differences in consecutive RR-intervals, called RR-increments, may be candidates for tools providing insight into this dynamics. Heart rhythm statistics based on RR-increments indicate the dynamical profile of RR-intervals. These statistics, together with their evolution over time after HTX, serve as the dynamical landscape for long-term HTX recipients. An additional benefit is that the method allows us to discern the appearance of erratic heart rhythm dynamics.

In the following, we hypothesize that an increase in certain HRV indices indicating short-term variability discriminates HTX patients who have a history of aspergilliosis and/or rejections from HTX patients whose overall clinical state is free of complications. In particular, we show that these indices, based on RR-increments, increase with the passing of time after the surgery in HTX patients with complications, while in the case of the stable HTX patients, they do not show any change.

Recently, it has been found that so-called fragmentation indices may lead to a better assessment of the dynamics of heart rate and also facilitate the detection of erratic heart rhythm (Costa et al., 2017). The fragmentation indices characterize short sequences with certain dynamical patterns, such as occurrences of successive accelerations or successive decelerations. In the following, we propose to include entropic measures for the fragmentation, as entropic measures collect and summarize the properties discovered by the fragmentation indices.

Entropic measures, such as approximate entropy or sample entropy, have been used in HRV estimates for more than 20 years (Pincus, 1991; Richman and Moorman, 2000). The novelty of our approach consists of applying them to RR-increments instead of RR-intervals. Accordingly, we also propose a modification of the fragmentation approach. Our concept of fragmentation includes zero value RR-increments. This extension is important because in the case of heart rhythms of HTX patients, we observe an abundance of zero RR-increments (Makowiec et al., 2013, 2014).

### 1.1. Outline

The rest of this article is organized as follows. Section 2 describes in detail the methods used. Starting with a description of the group of patients and the signals considered, we explain the concept of the dynamical landscape, and define the entropic measures based on Shannon entropy. Also, we present standard indices used in estimates and introduce some fragmentation indices. Our novel results are presented in section 3 and discussed in section 4. Finally, section 5 gives the conclusions.

## 2. Methods

### 2.1. Subject data

#### 2.1.1. Group of patients

Patients who had received HTX in the Cardiosurgery Department of the Medical University of Gdansk, Poland, were eligible for the study. All the patients in the HTX group were receiving standard immunosuppressive therapy. Beta-blocker therapy was a part of the treatment during the follow-up period. At the time of the measurements, the patients had to be in good physical condition without echocardiographic signs of acute rejection, heart failure or left ventricular dysfunction. The following exclusion criteria were applied: a history of pacemaker implantation, non-sinus rhythm, clinically unstable condition, less than 3 recordings during the time of the observations, and unwillingness to participate in Holter monitoring. We also excluded ECG Holter recordings with frequent ventricular and supraventricular arrhythmia and more than 10% artifacts.

Depending on the progress of the HTX recipient after surgery: *progress free of complications* or *progress not free of complications*, the patients were divided into the two groups denoted as F and NF, respectively. In Table 1, a clinical description of the participants in both groups is given. The group of patients free of complications consisted of six subjects with 24 signals. The signals were identified by the patient's ID and the number of months since the surgery: *F*_1_ (12, 20, 67), *F*_2_ (14, 19, 96), *F*_3_ (14, 36, 50, 61, 86), *F*_4_ (12, 46, 57), *F*_5_ (8, 12, 24, 36, 66, 78), and *F*_6_ (8, 12, 24, 36). The group of *NF* patients who experienced complications after HTX consisted of eight subjects with 34 signals, and was arranged in the same way as the *F* group: *NF*_1_ (14, 24, 37), *NF*_2_ (20, 24, 25, 26, 36, 63), *NF*_3_ (6, 12, 50, 64, 75), *NF*_4_ (6, 32, 39, 91), *NF*_5_ (9, 12, 19, 36, 61), *NF*_6_ (7, 17, 36), *NF*_7_ (12, 63, 78), *NF*_8_ (14, 23, 38, 39, 42).

**Table 1:** Patient demographic data.

**Patient ID**	**Sex**	**Age at HTX**	**Year/method of HTX**	**Diagn. before HTX**	**Graft vasculopathy**	**HT**	**DM**	**Ch RF**	**2R**	**C M V**	**Stroke**	**1st year FI**	**LVEF %graft echo**	**Outcome**
**HTX RECIPIENTS: PROGRESS WITH COMPLICATION**														
*NF*_1_	M	65	2008/	CAD	0	1	1	1	**1**	1	1	**0**	60	Survival
			bicav											
*NF*_2_	M	47	2009/	CAD	1	1	1	1	**1**	0	0	**0**	45	Death
			bicav											
*NF*_3_	M	59	2011/	CAD	0	1	1	0	**1**	0	1	**1**	65	Survival
			bicav											
*NF*_4_	F	36	2009/	myocarditis	0	1	0	1	**1**	0	0	**0**	65	Survival
			biatr											
*NF*_5_	F	55	2010/	DCM	0	1	1	0	**1**	0	0	**0**	65	Survival
			bicav											
*NF*_6_	M	33	2013/	DCM	0	1	1	0	**0**	0	1	**1**	60	Survival
			bicav											
*NF*_7_	F	57	2009/	CAD	1	1	1	1	**1**	0	0	**0**	60	Death
			biatr											
*NF*_8_	M	51	2010/	DCM	0	1	1	0	**0**	1	0	**1**	60	Survival
			bicav											
**HTX RECIPIENTS: PROGRESS FREE OF COMPLICATIONS**														
*F*_1_	M	52	2011/	CAD	0	0	0	0	**0**	0	0	**0**	65	Survival
			bicav											
*F*_2_	M	51	2008/	CAD	0	1	0	0	**0**	0	0	**0**	65	Survival
			biatr											
*F*_3_	M	55	2009/	CAD	0	0	0	0	**0**	0	0	**0**	60	Survival
			biatr											
*F*_4_	M	49	2012/	DCM	0	1	0	0	**0**	0	0	**0**	57	Survival
			bicav											
*F*_5_	M	24	2010/	DCM	0	0	0	0	**0**	0	0	**0**	65	Survival
			bicav											
*F*_6_	M	61	2009/	CAD	0	0	0	0	**0**	0	0	**0**	60	Survival
			biatr											

*CAD, coronary artery disease; DCM - dilated cardiomyopathy; HT, hypertension; DM, diabetes mellitus; ChRF, chronic renal failure; 2R, graft rejection; CMV, cytomegalovirus infection; FI, fungal infection; bicav, bicaval heart transplant; biatr, biatrial heart transplant. The bold type indicates diseases discriminative for the NF group: HTX with complications*.

#### 2.1.2. Control group

The control group was constructed from the pool of healthy people described in Makowiec et al. (2015a), whose age matched the age of the HTX patients considered. In total, this group consisted of 12 women and 15 men, aged 60–69. All the subjects passed routine tests for an overall healthy state. All the subjects gave their written, informed consent, which was approved by the Ethics Committee of Medical University of Gdańsk.

#### 2.1.3. Signals studied and their preprocessing

Twenty-four-hour ECG Holter recordings were analyzed on a Del Mar Reynolds system (Spacelabs Healthcare, United States). The sampling rate of ECG was 128 Hz, which ensured 8 ms accuracy for the times of identification of R-peaks of the QRS complex. The quality of the ECG recordings and accuracy of R-peak detection were verified by visual inspection by experienced cardiologists. All normal beats were carefully annotated, so that only normal sinus rhythms were considered in our investigations. The period of nocturnal rest was discerned individually, in each recording separately, according to the appearance of consecutive hours with a low heart rate. The rate analysis was based on one-hour average windows. Each signal started at the hour with the slowest heart rhythm. This means that we can assume that parts with the strongest transitions in ANS due to the sleep cycle are included. Since erratic rhythms tend to be episodic, longer recordings (in terms of hours) had to be considered.

Each signal was edited to preserve RR-intervals between normal-to-normal beats only. Short segments, consisting of less than 5 values, with artifacts or not normal beats were substituted by the medians estimated from the last seven normal beats. Longer segments with wrong data were deleted, which was annotated correspondingly in the time-lapse data. Hours with an overall quantity of normal-to-normal beats of less than 95% were excluded from further analysis. Finally, twenty thousands subsequent beats were removed, starting at the beginning of the nocturnal period.

### 2.2. Dynamical landscape method

#### 2.2.1. Method description and motivation

**Figure 1:**
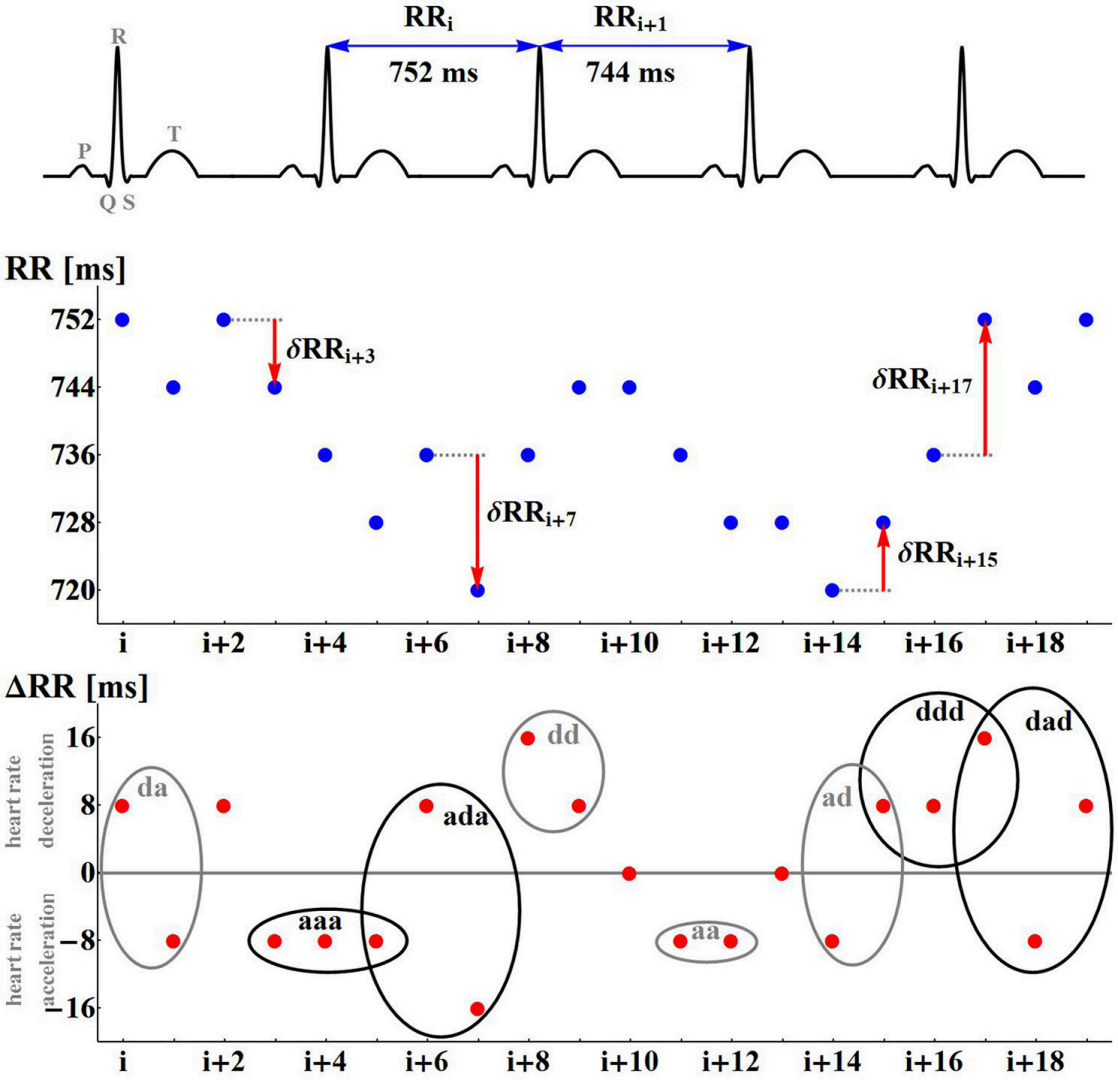
Illustrations of the nomenclature and concepts used. The differences δ*RR* are calculated for the numbered RR-intervals extracted from the ECG recording. A positive difference is encoded as *d*; a negative difference is encoded as *a*. A signal δ*RR* is represented as signals of symbols *d* and *a*.

A non-linear system approach provides a variety of methods which can reveal dynamical preferences of a system based on time series (see Bradley and Kantz, 2015 for the latest review). In the case of cardiac dynamics represented by RR-intervals (see Figure 1), time intervals between successive heart contractions, a popular idea is to associate symbols with values of the heart rhythm and then quantify dynamical preferences of the RR-interval signal by regularities/irregularities in the symbolic signal representation (Sassi et al., 2015). The simplest example of symbolization follows heart rhythm accelerations *a* and decelerations *d*. Then the dynamics is analyzed according to short-term pattern representations of time sequences (see Figure 1, bottom plot).

Having extracted successive RR-intervals (see Figure 1 the middle line plot):

$$\text{R}\text{R}:RR_{0},RR_{1},RR_{2},\ldots ,RR_{N}$$

the sequence of differences between consecutive RR-intervals, called RR-increments, is derived:

$$\Delta \text{RR}:\delta RR_{1},\delta RR_{2},\ldots ,\delta RR_{N}$$

with δ*RR_i_* = *RR_i_* − *RR*_*i*−1_ for *i* = 1, …, *N*. Each δRR_i_ positive denotes a heart rate deceleration, while δ*RR_i_* negative means a heart rate acceleration. When δ*RR_i_* equals zero, it must be assumed that there is no change in the heart rate dynamics.

It is of note that the classification of any RR-increment into deceleration, acceleration or zero-change, depends on the signal resolution. We found that our Holter system resolution of 8 ms was fine enough to filter out the noise HRV from the system HRV. However, in a general case, when the resolution is smaller, one should consider a tolerance ε to associate RR-increments which in modulus are lower than ε with the zero class and then, accordingly, group the other RR-increments into classes labeled according to the desired resolution. For example, if the resolution equals to 1 ms, the resolution of 7 ms can be achieved by the following grouping {…, (−10, −9, −8, −**7**, −6, −5, −4), (−3, −2, −1, **0**, 1, 2, 3), (4, 5, 6, **7**, 8, 9, 10), …}, where values in bold are labels for the subsequent classes {…, −7, 0, 7, …}.

In our case, all the recordings were at the 8 ms resolution and therefore all the signals were preprocessed with this discretization. Application of the symbolization described above, in place of a sequence of RR-increments (e.g.,{…, 24, 0, −8, −8, 16, 0, … }) provides a sequence of symbols ({…*d*, 0, *a*, *a*, *d*, 0, … }, respectively). In this way, each RR-increment is represented by one of the three symbols, elements of the set *S* = {*a*, *d*, 0}. The set of symbols *S* will be called the space of actions, while its elements will be called actions. A variety of single actions: *d*, *a*, 0, a variety of patterns of two-consecutive actions: *dd*, *da*, *ad*, *aa*, *d*0, 0*d*, *a*0, 0*a*, 00, and in general, a variety of *L*-consecutive actions, is the source for quantification of signal dynamics.

If one takes into account not only the sign of an RR-increment but also its magnitude, a significantly larger space of actions than {*a*, *d*, 0} can be constructed. In the case of RR-intervals recorded with 8 ms accuracy, the largest action space is the space constructed at the signal recording accuracy, and it consists of actions *S* = {…, −16, −8, 0, 8, 16, … }, which are delimited by the largest acceleration on the left and the largest deceleration on the right. This means that the actions are symbols representing RR-increment values. Consequently, it is possible to investigate the probability distributions for a single action, i.e., for the appearance of a given RR-increment, for the presence of 2 given consecutive RR-increments, or in general *L* consecutive RR-increments. Note that with different scaling of the space of actions, it is possible to observe the dynamics at another scale. Namely, by manipulating the resolution, it is possible to estimate variability related to events at a certain scale.

Irregularities in short timescales are investigated with so-called short-term HRV indices. These are standard indices such as RMSSD, pNN50 or HF, which are well-recognized measures of short-term variability (Task Force, 1996; Goldberger and Stein, 2015). In general, irregularities within segments consisting of less than ten RR-intervals are assumed to describe short-term HRV (Sassi et al., 2015). It is known that if the patient is predominantly in normal sinus rhythm, such variability can be interpretable for ANS assessment purposes (Goldberger and Stein, 2015).

The method considered here, called the dynamical landscape of long-term HTX recipients, includes the group of estimators of short-term HRV. It extracts and then qualifies heart period dynamics according to the following three aspects: (A) RR-increments, (B) certain statistics of RR-increments, and (C) changes in statistics with the passing of time.

In particular, we concentrate on information obtained from statistics of short segments of RR-increments by Shannon entropy (Shannon, 1948) and concepts related to Shannon entropy concepts describing stochastic dynamics. However, different statistics of RR-increments could be considered for aspect (B). We will work with standard HRV indices to obtain the reference point. Results provided by entropic tools will be compared with fragmentation heart rate indices (Costa et al., 2017)—a novel group of estimators of short-term HRV, which is based on symbolic dynamics of accelerations and decelerations.

#### 2.2.2. Short-term HRV indices studied

**Figure 2:**
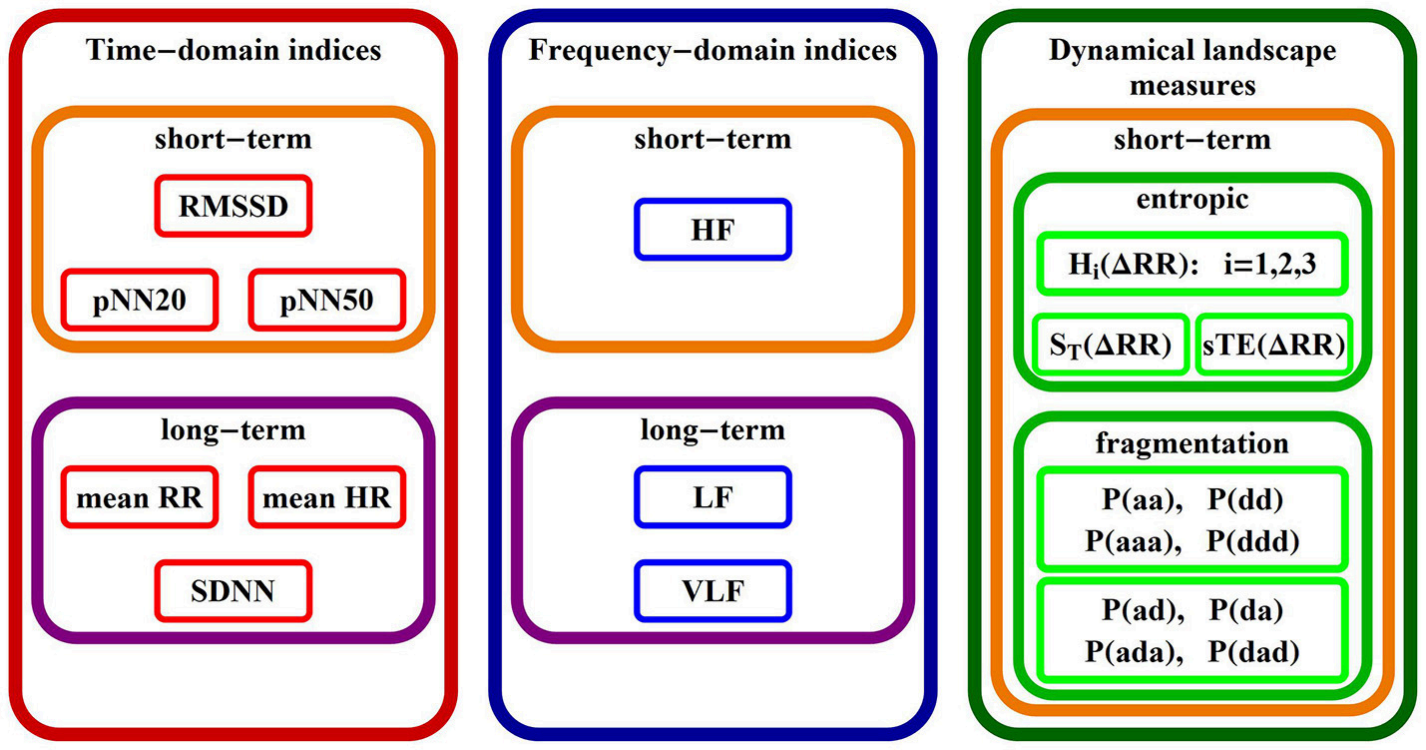
Table of HRV indices: standard and new ones, considered in the paper.

In Figure 2 we give a list of names of all indices studied in the rest of the paper, grouped according to the commonly used classes (Task Force, 1996). Below, we introduce/explain them.

**(a) Standard HRV indices**

From the set of standard HRV indices (Task Force, 1996; Goldberger and Stein, 2015; Sassi et al., 2015), we decided that the best information about the overall variability and short-term variability of the signals presented would be gained by the following measures:

- mean HR - heart rate in [1/min];
- Mean HR is strongly advised to be the first number to be evaluated to give the rest of the values some context. As we consider nocturnal recordings, we expect to observe the slowest rhythm of the circadian cycle.
- SDNN - standard deviation of RR-intervals in [ms];
- SDNN is said to capture the total HRV. It estimates the variation of a signal with respect to the mean signal value. In the case of cardiac patients, low values of SDNN (<50) have been associated with a markedly higher risk of mortality (Nolan et al., 1998).
- VLF - very low frequency power in [ms^2^];
- VLF captures the magnitude of underlying oscillations in RR-intervals of periods between 25 s to 5 min (from 0.003 to 0.04 Hz). In healthy adults, VLF appears to reflect vagal activity since it is abolished by atropine administration and unaffected by beta-blockade (Goldberger and Stein, 2015). It also appears to reflect the activity of the renin-angiotensin system, since it is reduced by ACE inhibition (Taylor et al., 1998; Tripathi, 2011). In contrast, VLF power is increased by sleep abnormalities such as sleep disordered breathing events, which should be taken into account in the case of our nocturnal recordings. Decreased VLF power has been shown to be strongly related to adverse outcomes (Goldberger and Stein, 2015).
- RMSSD - the square root of the mean of the sum of the squares of δ*RR* in [ms];
- RMSSD is said to refer to vagal tone, as it directly estimates variation among consecutive RR-intervals, avoiding the mean of a signal as the reference value (Kleiger et al., 2005).
- pNN50 - the percentage of |δ*RR*| greater than 50 ms;
- pNN50 captures activity of the vagal system as it accounts for large accelerations or decelerations, which are assumed to result from high activity of the vagal system.
- HF spectral power in oscillations between 0.15 Hz and 0.40 Hz in [ms^2^];
- HF in the case of sinus rhythm reflects modulation of efferent vagal activity by respiration (respiratory sinus arrhythmia). For a subject in the supine position, the HF spectrum has a peak corresponding to the predominant respiratory frequency.
- LF spectral power in oscillations of 0.04 and 0.15 Hz in [ms^2^];
- LF is assumed to describe the combined modulation of vagal and sympathetic activity, which is often related to the baroreflex feedback loop (Goldstein et al., 2011).
- pNN20 - the percentage of |δ*RR*| greater than 20 ms;
- pNN20 is supposed to provide enhanced discrimination between a variety of normal and pathological conditions (Mietus et al., 2002), especially in the case of HTX patients, when the overall variability is low.

**(b) Entropic measures**

It is said that the occurrence of an action *i* carries an uncertainty which should be quantified as ln (1/*p*(*i*)) (Shannon, 1948; Kaiser and Schreiber, 2002). So the smaller the probability is of observing an action *i*, the larger the uncertainty related to this action is. Averaging uncertainty over all actions, we obtain the simplest tool for quantification of the variety of actions in a system represented by a given signal. This tool is called Shannon entropy (Shannon, 1948). It occurs that for more predictable signals, the Shannon entropy is lower. In particular, Shannon entropy attains the lowest value (zero) when a signal is completely predictable, while the maximal value entropy (logarithm of the number of states in *S*) is attained when all actions occur with the same probability.

Let *S_L_* denote the state space of *L* consecutive actions of a given RR-interval signal. Let *i* denote any RR-increment from the space *S*_1_, (*i*, *j*) ∈ *S*_2_ stand for 2 consecutive RR-increments and (*i*, *j*, *k*) ∈ *S*_3_ stand for 3 consecutive RR-increments. Subsequently, for each Δ**RR** signal, we quantify its properties by the following entropic measures:

- entropy of a single action encoded by RR-increment:
  (1) $$\begin{array}{l} H_{1}\left(\Delta \text{RR}\right)=-\underset{i\in S_{1}}{\sum }p\left(i\right)\ln\;p\left(i\right) \end{array}$$
- entropy of a pair of successive RR-increments:
  (2) $$\begin{array}{l} H_{2}\left(\Delta \text{RR}\right)=-\underset{\left(i,j\right)\in S_{2}}{\sum }p\left(i,j\right)\ln\;p\left(i,j\right) \end{array}$$
- entropy of a triplet of successive RR-increments:
  (3) $$\begin{array}{l} H_{3}\left(\Delta \text{RR}\right)=-\underset{\left(i,j,k\right)\in S_{3}}{\sum }p\left(i,j,k\right)\ln\;p\left(i,j,k\right). \end{array}$$

Moreover, so-called *excess entropy*, defined as *h_i_* = *H_i_* − *H*_*i*−1_ for *i* = 2, 3, … , measures the increase of entropy when an extra RR-increment precedes the given sequence of RR-increments (Hlavackova-Schindler et al., 2007). In the case of dynamical series, the excess entropy *h*_2_ is known to be the best estimator of the *entropy of transition rates S_T_*, i.e.,:

(4) $$\begin{array}{l} S_{T}(\Delta RR)=H_{2}(\Delta RR)-H_{1}(\Delta RR). \end{array}$$

*S_T_* evaluates the system dynamics as if it were a Markov chain (Ciuperca and Girardin, 2005), i.e., memoryless dynamics governed by a table of transition probabilities. It has been proved that *S_T_* is equal to approximate entropy (ApEn) (Pincus, 1991), a popular metrics used in assessment of signals with RR-intervals.

Clearly, other excess entropies can also be of interest. If these entropies drop when the length of a sequence of RR-increments grows, the process is regular and predictable. Conversely, a constant value of *h_i_*, called the entropy rate, suggests that each new action is not completely predictable (Sassi et al., 2015). The entropy rate is a basic characterization of Markov chain dynamics.

*Self-transfer entropy* (sTE)—a concept proposed by Schreiber (2000), and considered further in Kaiser and Schreiber (2002), to measure the coupling between any two interacting systems, applied in a way that accounts for the influence of the past on the current action, which can be expressed as the following difference:

(5) $$\begin{array}{l} sTE\left(\Delta \text{RR}\right)=h_{3}\left(\Delta \text{RR}\right)-h_{2}\left(\Delta \text{RR}\right). \end{array}$$

sTE describes whether a simplified model, represented as a Markov chain, is coupled to its past. So, sTE estimates memory effects, which are not encoded in a transition matrix of the Markov chain model.

Finally, let us underline that if RR-increments occur in a signal independently of each other, *H*_2_ = 2*H*_1_, *H*_3_ = 3*H*_1_. These relations lead to *S_T_* = *H*_1_ and *sTE* = 0, which means that the Markov chain model with the transition matrix driven by the distribution of single actions describes completely the dynamics of RR-increments.

**(c) Heart rate fragmentation indices**

As the vagal activity results in rapid changes in heart rhythm, it can be hypothesized that smoothness of the signal, i.e., consecutive accelerations or consecutive decelerations, can be related to the vagal modulation (Costa et al., 2017). Conversely, alternations between accelerations and decelerations are attributed to non-vagally mediated regulatory mechanisms. These properties could be directly linked to short-term variability, precisely to the presence or absence of a particular type of *L*-symbol clusters. It has been shown that the participation of abrupt changes in the sign of RR-increments increases with age (Costa et al., 2017). The corresponding metrics, called heart rate fragmentation indices, have been proposed as markers of the integrity of the regulatory network of heartbeats.

Pursuing this idea, we investigate the occurrence of patterns which have a clear dynamical classification. Namely, if *a* denotes an acceleration, and *d* denotes a deceleration, we estimate probabilities of the following events (see Figure 1):

- segments describing monotonic increases or decreases:
  - P(aa): probability of two successive accelerations: (δ*RR*_i_ < 0, δ*RR*_i+1_ < 0)
  - P(dd): probability of two successive decelerations: (δ*RR*_i_ > 0, δ*RR*_i+1_ > 0)
  - P(aaa): probability of three successive accelerations: (δ*RR*_i_ < 0, δ*RR*_i+1_ < 0, δ*RR*_i+2_ < 0)
  - P(ddd): probability of three successive decelerations: (δ*RR*_i_ > 0, δ*RR*_i+1_ > 0, δ*RR*_i+2_ > 0)
- segments describing alternations in dynamics;
  - P(ad): probability of two alternates, acceleration first (δ*RR*_i_ < 0, δ*RR*_i+1_ > 0)
  - P(da): probability of two alternates, deceleration first (δ*RR*_i_ > 0, δ*RR*_i+1_ < 0)
  - P(ada): probability of three alternates, acceleration first (δ*RR*_i_ < 0, δ*RR*_i+1_ > 0, δ*RR*_i+2_ < 0)
  - P(dad): probability of three alternates, deceleration first (δ*RR*_i_ > 0, δ*RR*_i+1_ < 0, δ*RR*_i+2_ > 0)

The quantities defined above, when grouped appropriately, provide approximate estimates for the fragmentation indices of Costa et al. (2017). They approximate these only if we exclude the zero RR-increments from the counts. Subsequently,

– PIP, percentage of abrupt changes in the sign of RR-increments, can be estimated as

PIP=P(ad)+P(da)

– PSS, complement to the percentage of short RR-intervals in monotonic sequences, can be estimated as

PSS=1-[P(aaa)+P(ddd)]

– PAS, percentage of alternative *L*-clusters, in the case of *L* = 3 is

PAS=P(ada)+P(dad).

Quantities from the above list influence the Shannon entropy value selectively. However, by summing up all of these particular ingredients, we gain the total measure of the difference between the actual probability distribution and the flat distribution where all events have the same probability of occurring.

### 2.3. Statistical analysis

The counts of events were performed with our own software (C++ language). The entropy calculations, as well as fragmentation indices and standard HRV indices, were processed with MATLAB R2016b (MathWorks Inc.). As the normality test (Shapiro-Wilk) was not passed for data pooled in the patient groups *F* and *NF* (*p* < 0.05), differences between the groups were estimated by Kruskal-Wallis One-way ANOVA on ranks. The t-test for paired data (Wilcoxon Signed Rank Test if normality failed) was performed on groups in order to discover the importance of the dynamics. Additionally, with logistic regression (Bewick et al., 2005), we investigated the classification abilities of the HRV measures investigated: free of complications vs. with complications. The linear regression estimates were used in the evaluation of the evolution of indices. The tests were performed with One-way ANCOVA. Sigma Plot 13.0 (Systat Software, Inc) was utilized in all the tests described above.

As the set of data is rather small in size, a leave-one-subject-out cross-validation technique was used to verify the robustness of the correlation coefficient estimates. This means that N-1 (out of N) patients in a group were used to estimate the global regression coefficient. This procedure was carried out with exclusion of each subject in each group. It was observed that the values for the regression remained appreciably similar when different subjects were selected in estimates.

## 3. Results

### 3.1. Dynamical landscape resulting from standard HRV indices

Table 2 presents a descriptive characterization of the standard indices of short-term HRV values studied. We show indices describing the overall variability of signals to find the general message which can be derived from standard indices estimated from the signals considered. The indices of short-term variability are presented together: RMSSD, pNN50, pNN20, and HF (in bold).

**Table 2:** Descriptive statistics and linear regression analysis for typical HRV indices.

**Index symbol**	**HTX F**	**HTX NF**	***p*-value**	**Healthy sexagenarians**
**(a) Descriptive statistics: median [25%, 75%] and *p*-value (in bold in case *p* < 0.05) for difference in medians between the HTX groups**				
HR	85.4 [75.6, 93.9]	79.6 [70.4, 86.4]	**0.038**	60.6 [57.8, 67.8]
RR	703 [641, 798]	757 [695, 862]	**0.048**	963 [892, 1,051]
SDNN	29.6 [21.7, 45.9]	29.0 [21.4, 54.8]	0.912	73 [61, 86]
**RMSSD**	8.31 [7.45, 8.95]	10.9 [7.91, 21.0]	**0.001**	25 [21, 37]
**pNN20**	1.22 [0.37, 2.27]	6.31 [0.89, 16.5]	**0.001**	40 [29, 54]
**pNN50**	0.025 [0.001, 0.064]	0.083 [0.036, 2.05]	**0.001**	3.6 [2.1, 13]
VLF	736 [329, 1345]	567 [320, 1332]	0.825	4,575 [3127, 7253]
LF	2.8 [1.2, 7.3]	6.63 [3.07, 61.0]	**0.003**	477 [317, 717]
**HF**	13.0 [8.6, 17.2]	27.03 [10.2, 101.9]	**0.007**	207 [137, 364]
**(b) Regression analysis: slope ± stderr (*R*, *p*-value) for the given HTX group dependence on time after surgery (in bold regression which is statistically significant at *p* < 0.05), and *p*-value (in bold in case p < 0.05) for difference in the regressions between the HTX groups**				
HR	−0.43 ± 1.0 (−0.09, 0.679)	−1.47 ± 0.91 (−0.27, 0.117)	0.451	
RR	4.7 ± 9.6 (0.10, 0.629)	15.0 ± 10.2 (0.25, 0.150)	0.467	
SDNN	1.68 ± 1.50 (0.23, 0.274)	**9.02 ± 3.02 (0.47, 0.005)**	**0.044**	
**RMSSD**	0.03 ± 0.12 (0.05, 0.790)	**12.2 ± 3.93 (0.48, 0.004)**	**0.007**	
**pNN20**	0.110 ± 0.179 (0.13, 0.545)	**3.49 ± 1.11 (0.48, 0.004)**	**0.008**	
**pNN50**	−0.002 ± 0.017(0.00, 0.924)	**1.47 ± 0.48 (0.48, 0.004)**	**0.007**	
VLF	34.3 ± 102 (0.07, 0.738)	291 ± 188 (0.26, 0.131)	0.258	
LF	0.39 ± 0.72 (0.12, 0.587)	**91 ± 35 (0.42, 0.014)**	**0.021**	
**HF**	−0.19 ± 0.72 (0.05, 0.799)	**631 ± 245 (0.41, 0.015)**	**0.022**	

*(a) Difference between groups by one-way ANOVA on ranks (normality failed) results: medians [25%, 75%], and p-value for the difference between groups*.

*(b) Linear regression estimates with respect to age: linear regression coefficient ± StdErr (R, p-value: of the test H_0_: slope = 0, H_1_: slope ≠ 0), and p-value of the test H_0_: equal slopes, H_1_: different slopes, provided by one-way ANOCOVA*.

Section (a) of the table provides a static characterization of the signals of all the groups studied, including characterization of healthy coevals. We see that the values of the standard HRV indices obtained for the groups of the HTX patients are strongly distinct from the values obtained for healthy people in their sixties. In general, the heart rhythm of HTX patients is significantly faster than the rhythm of healthy persons. Indices pNN20, pNN50 and all spectral indices show the most striking difference. The healthy population provides many times larger values for these measures. We can also see that all short-term HRV indices, namely RMSSD, pNN20, pNN50 and HF, discriminate (statistically significantly) the F group from the NF group. In all cases, the results of the NF group are higher than those of the F group. Logistic regression analysis reveals that RMSSD and pNN20 provide for a statistically validated classification of the F and NF group: 18/19 signals in the F group and 25/24 signals in the NF group were properly classified by RMSSD/pNN20, respectively.

Section (b) of the table presents results of the evolution over time of all the indices studied, obtained by the regression analysis performed on the F group and the NF group. It occurs that all short-term HRV indices obtained for the NF group exhibit growth with time after HTX. These regressions are statistically significant. This property does not hold for the indices found for the F group. Here, no significant relation between the index value and the passing of time has been found. Moreover, the One-way ANCOVA test for equal slopes in both groups failed for all short-term indices.

In conclusion, the standard indices of short-term HRV prove the difference between the groups F and NF consisting of both the value of the index (higher in NF) and the tendency to change over time (growing in NF).

**Figure 3:**
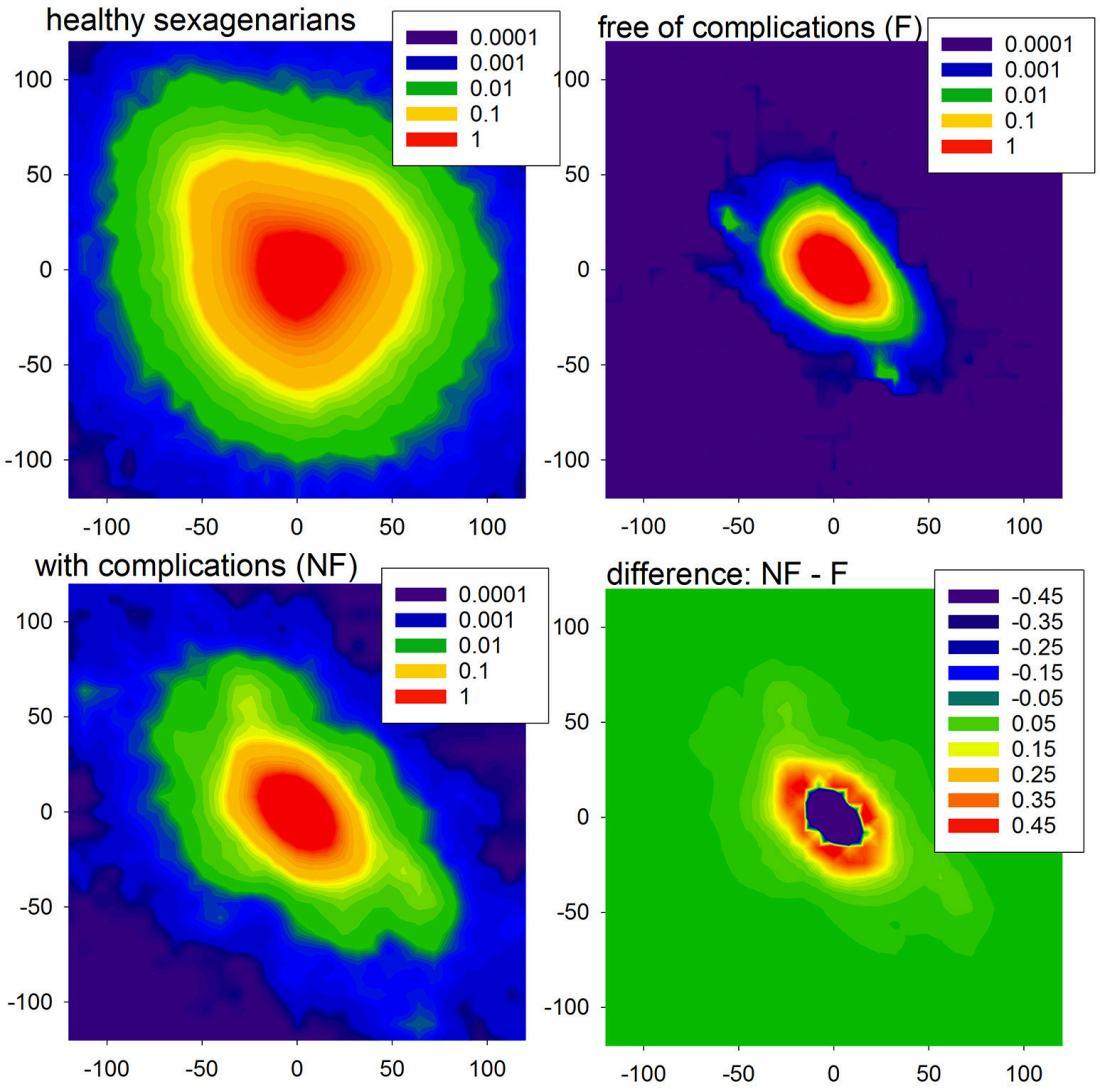
The probability density (in %, log-scale) of a pair of events in the case of healthy sexagenarians **(top-left)**, HTX patients free of complications **(top-right)**, and with complication **(bottom-left)**. Note that the scales are different. Finally, the difference between density of the groups NF and F is shown **(bottom-right)**.

The entropic measures quantify the system dynamics based on the probability distribution of events, here actions with accelerations or decelerations. In Figure 3, we show the mean probability of a pair of actions obtained from signals of healthy sexagenarians and the mean of the HTX patients, divided into the F and NF groups. We also show the difference in two-point distributions between the NF and F groups. The bottom line describes the first RR-increment of a pair. The vertical line corresponds with the second RR-increment. All the plots display a kind of symmetry with respect to the diagonal NW – SE (NW for North-West and SE for South-East are geographic map directions). This shows that after any acceleration, a deceleration of the similar size is the most probable event. It is noticeable that in the case of healthy people, after any deceleration there is not such a strong prevalence for an acceleration as there is in the case of the HTX patients. There is an evident difference between healthy people and HTX patients from the F group, reflected in the sizes of accelerations and decelerations. In the case of the signals from the F group, most changes are within ± 50 ms, which is much less than the spread of changes in healthy people. These observations suggest that the signals of HTX patients are significantly more predictable than those of healthy people. The probability distributions obtained from patients in the NF group initially resemble the distribution of healthy coevals. However, closer examination reveals that the NW–SE spread is larger than in the plot of healthy people. It is also very different from the distribution estimated from signals of patients in the F group. This difference is shown in Figure 3, bottom-right plot.

A descriptive characterization of the values of entropic measures is given in section (a) of Table 3. It shows that all entropic measures *H*_3_, *H*_2_, *H*_1_, *S*_*T*_, and *sTE* take values considerably lower than levels obtained for the corresponding healthy coevals. It is noticeable that the median value obtained from the HTX patients pooled in the NF group is higher than the median obtained for the F group. In each case, with the exception of *sTE*, the difference has a high statistical significance. Logistic regression analysis additionally ensures that all the entropic measures provide a statistically satisfactory separation between the groups studied. The best classification abilities are found for sTE and H1 which, when applied simultaneously, provided a proper classification of 19 signals in the F group and 27 signals in the NF group.

**Table 3:** Descriptive statistics and linear regression analysis for entropic measures.

**Index symbol**	**HTX F**	**HTX NF**	***p*-value**	**Healthy sexagenarians**
**(a) Descriptive statistics: median [25%, 75%] and *p*-value (in bold in case *p* < 0.05) for difference in medians between the HTX groups**				
*H*_3_	3.80 [3.42, 3.97]	4.60 [3.90, 5.51]	**<0.001**	7.32 [6.61, 7.89]
*H*_2_	2.63 [2.44, 2.80]	3.20 [2.64, 3.90]	**<0.001**	5.02 [4.53, 5.69]
*H*_1_	1.38 [1.29, 1.47]	1.67 [1.37, 2.11]	**0.001**	2.53 [2.31, 2.90]
*S*_*T*_	1.25 [1.11, 1.32]	1.51 [1.27, 1.83]	**0.002**	2.49 [2.24, 2.72]
*sTE*	0.10 [0.07, 0.14]	0.13 [0.08, 0.21]	0.195	0.24 [0.17, 0.45]
**(b) Regression analysis: slope ± stderr (*R*, *p*-value) for the given HTX group dependence on time after surgery (in bold regression which is statistically significant at *p* < 0.05), and *p*-value (in bold in case *p* < 0.05) for difference in the regressions between the HTX groups**				
*H*_3_	0.007 ± 0.04 (0.04, 0.849)	**0.35 ± 0.10 (0.51, 0.002)**	**0.006**	
*H*_2_	0.011 ± 0.03 (0.08, 0.689)	**0.26 ± 0.08 (0.52, 0.002)**	**0.005**	
*H*_1_	0.008 ± 0.013 (0.13, 0.553)	**0.15 ± 0.04 (0.53, 0.001)**	**0.004**	
*S*_*T*_	0.003 ± 0.014 (0.04, 0.830)	**0.12 ± 0.04 (0.50, 0.003)**	**0.008**	
*sTE*	0.006 ± 0.005 (0.27, 0.196)	**0.034 ± 0.011 (0.47, 0.005)**	**0.035**	

(a) Difference between groups by one-way ANOVA on ranks (normality failed) results: medians [25%, 75%], and p-value for the difference between groups.

*(b) Linear regression estimates for age: linear regression coefficient ± StdErr (R, p-value of the test H_0_: slope = 0, H_1_: slope ≠ 0), and p-value of the test H_0_: equal slopes, H_1_: different slopes, provided by one-way ANOCOVA*.

Finally, we performed tests for the presence of dynamical dependencies, i.e., the complexity of the signals collected in the groups F and NF. Subsequently we tested the hypothesis **H**_0_ : *S*_*T*_ < *H*_1_ vs. **H**_1_ : *S*_*T*_ ≥ *H*_1_, **H**_0_ : *sTE* = 0 vs. **H**_1_ : *sTE* ≠ 0, **H**_0_ : *H*_2_ < 2*H*_1_ vs. **H**_1_ : *H*_2_ ≥ 2*H*_1_, and **H**_0_ : *H*_3_ < 3*H*_1_ vs. **H**_0_ : *H*_3_ ≥ 2*H*_1_. Our data prove that all *H*_0_ can be rejected at a high significance level, for almost all tests *p* < 0.001, in both groups of patients. Hence the heart rate dynamics is not stochastic but involves cardiovascular properties.

**Figure 4:**
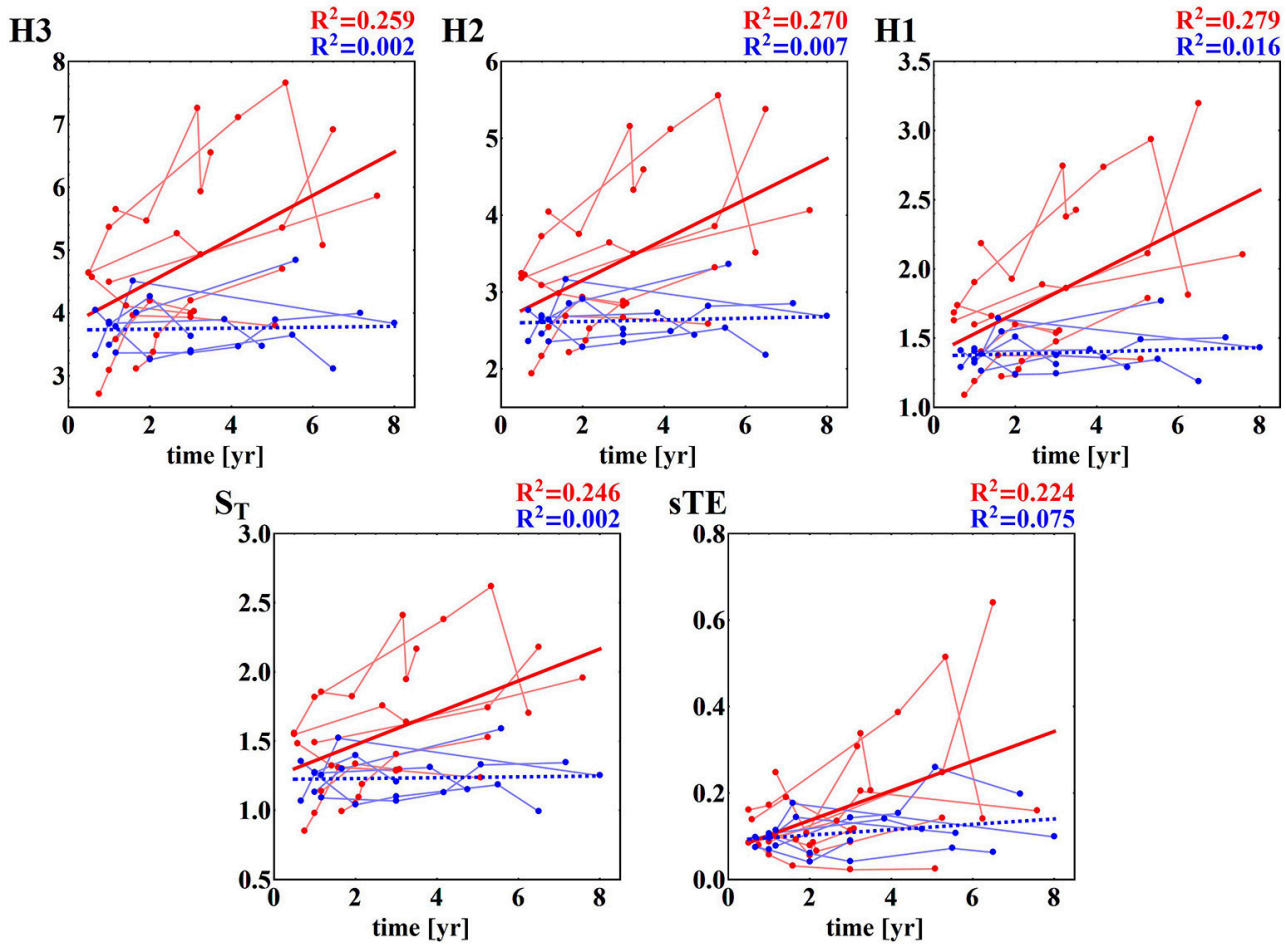
Time runs of the entropic measures estimated for RR-increments of individuals from HTX groups. Blue marks and curves refer to the F group of patients. Red notation is used for the NF patients. The bold curves represent the best linear regression approximation to the group data. The line is dashed if the hypothesis of the zero regression coefficient cannot be rejected. For both lines, R^2^ correlation coefficients are shown. Points corresponding to the same patient are connected by faded lines.

Furthermore, the HTX patients' signals pooled into the F group do not show any significant dependence on the time that has elapsed since the surgery, while the signals grouped in the NF class present a clear dependence on time—they grow as time progresses; see section (b) of Table 3. These dependencies on time are shown in subsequent panels of Figure 4. The regression coefficients *R*^2^ for entropies obtained from the NF group are not impressive, however they are high enough for the statistical validation of the linear growth of these entropies with time after the surgery. Note that this growth changes from patient to patient, and that there is one patient, *NF*_6_, in whom the growth is not observed. Note also that the highest speed of the growth exhibits the entropy *H*_3_, namely entropy which estimates the distribution of three RR-increments. In the case of the group of signals representing the F group, none of the entropies considered pass the test for non-zero increase with time. Therefore, we can hypothesize that there is no change in entropic measures in the signals of the F group.

### 3.2. Dynamical landscape resulting from measures of fragmentation

Fragmentation indices describe the system dynamics concentrating on three actions only: acceleration, deceleration and no change. RR-increments are filtered only by the definition of the no-change action. In our estimations, we assumed the same level—the level of signal resolution, for discerning the no-change action. The statistics of counts of actions (medians together with their 25th and 75th percentiles) of the fragmentation indices introduced by us are presented in Table 4. The indices are grouped into two classes corresponding to patterns of the monotonic dynamic and patterns of dynamics with alternations.

**Table 4:** Descriptive statistics and linear regression analysis for dynamical patterns indices.

**Index symbol**	**HTX F**	**HTX NF**	***p*-value**	**Healthy sexagenarians**
**(a) Descriptive statistics: median [25%, 75%] and *p*-value (in bold in case *p* < 0.05) for difference in medians between the HTX groups**				
**PATTERNS OF MONOTONIC DYNAMICS**				
*P*(*aa*)	2.84 [1.7, 4.3]	5.3 [2.2, 8.8]	**0.009**	17.1 [14.9, 18.5]
*P*(*dd*)	2.29 [1.8, 3.85]	4.9 [2.5, 7.7]	**0.004**	16.6 [12.2, 19.2]
*P*(*aaa*)	0.10 [0.03, 0.16]	0.36 [0.06, 1.2]	**0.004**	4.54 [3.17, 6.13]
*P*(*ddd*)	0.07 [0.03, 0.23]	0.33 [0.06, 0.75]	**0.006**	4.23 [2.61, 6.62]
**PATTERNS OF DYNAMICS WITH ALTERNATIONS**				
*P*(*ad*)	15.6 [14.4, 16.7]	17.3 [15.6, 21.5]	**0.002**	19.7 [16.8, 24.0]
*P*(*da*)	16.1 [15.1, 17.0]	17.5 [16.0, 21.6]	**0.006**	19.1 [16.2, 23.3]
*P*(*ada*)	7.32 [5.95, 8.41]	7.94 [6.60, 11.1]	0.060	6.45 [5.22, 1.01]
*P*(*dad*)	6.84 [5.65, 7.79]	8.14 [6.57, 10.6]	**0.007**	6.26 [4.89, 10.5]
**(b) Regression analysis: slope ± stderr (*R*, *p*-value) for the given HTX group dependence on time after surgery (in bold regression which is statistically significant at *p* < 0.05), and *p*-value (in bold in case *p* < 0.05) for difference in the regressions between the HTX groups**				
**PATTERNS OF MONOTONIC DYNAMICS**				
*P*(*aa*)	−0.01 ± 0.15 (0.00, 0.948)	**0.82 ± 0.31 (0.42, 0.013)**	**0.027**	
*P*(*dd*)	−0.08 ± 0.13 (0.13, 0.528)	**0.69 ± 0.26 (0.42, 0.013)**	**0.015**	
*P*(*aaa*)	−0.01 ± 0.02 (0.13, 0.557)	0.07 ± 0.06 (0.21, 0.240)	0.222	
*P*(*ddd*)	−0.02 ± 0.02 (0.23, 0.272)	**0.11 ± 0.05 (0.39, 0.022)**	**0.017**	
**PATTERNS OF DYNAMICS WITH ALTERNATIONS**				
*P*(*ad*)	0.20 ± 0.13 (0.32, 0.131)	**0.75 ± 0.34 (0.36, 0.034)**	0.160	
*P*(*da*)	0.26 ± 0.15 (0.34, 0.099)	**0.65 ± 0.32 (0.34, 0.048)**	0.299	
*P*(*ada*)	0.16 ± 0.14 (0.23, 0.271)	0.42 ± 0.28 (0.25, 0.144)	0.440	
*P*(*dad*)	0.11 ± 0.11 (0.21, 0.329)	0.27 ± 0.28 (0.17, 0.344)	0.626	

(a): Difference between groups by one-way ANOVA on ranks (normality failed) results: medians [25%, 75%], and p-value for the difference between groups.

*(b): Linear regression estimates for age: linear regression coefficient ± StdErr (R, p-value: of the test H_0_: slope = 0, H_1_: slope ≠ 0), and p-value of the test H_0_: equal slopes, H_1_: different slopes, provided by one-way ANOCOVA*.

First of all, let us admit the large distinction between values of indices describing the presence of the monotonic patterns in healthy sexagenarians and HTX patients, and the almost equivalent presence of the patterns corresponding to alternations in the dynamic. It occurs that persistent acceleration or deceleration of the heart after HTX is of many times lower probability than in a healthy organism. In the case of two-action patterns, this observation reveals the symmetry NW-SE of both probability distributions shown in Figure 3. This symmetry reflects the antipersistency in organization of accelerations and decelerations. However the sequences of accelerations and decelerations could occur with smaller steps than the signal resolution and therefore be hidden behind no-change actions. Referring to the two-element patterns *aa, dd, ad, da*, the values presented in section (a) of Table 4 allow an exact estimation of the role of the patterns of the no-change action. This calculation means that in the case of healthy sexagenarians, the statistics presented in the table cover 76.5% of all actions, while in the case of the F group of HTX patients, it represents only 36.8% actions, and in the case of the NF group of HTX patients, it represents 45% of actions.

### 3.3. Dynamical landscape resulting from entropic measures

Comparing fragmentation indices of the two HTX groups, F and NF, we see differences between the group values [statistically significant, *p* < 0.05, for all indices with the exception of *P*(*ada*)]. All indices show greater values for the NF group. The logistic regression test has proved that the groups are statistically satisfactorily separated by almost all standard statistical measures. The best classification abilities have been found for *dad* + *aaa*: 20 signals of the F group and 25 of the NF group were properly classified.

**Figure 5:**
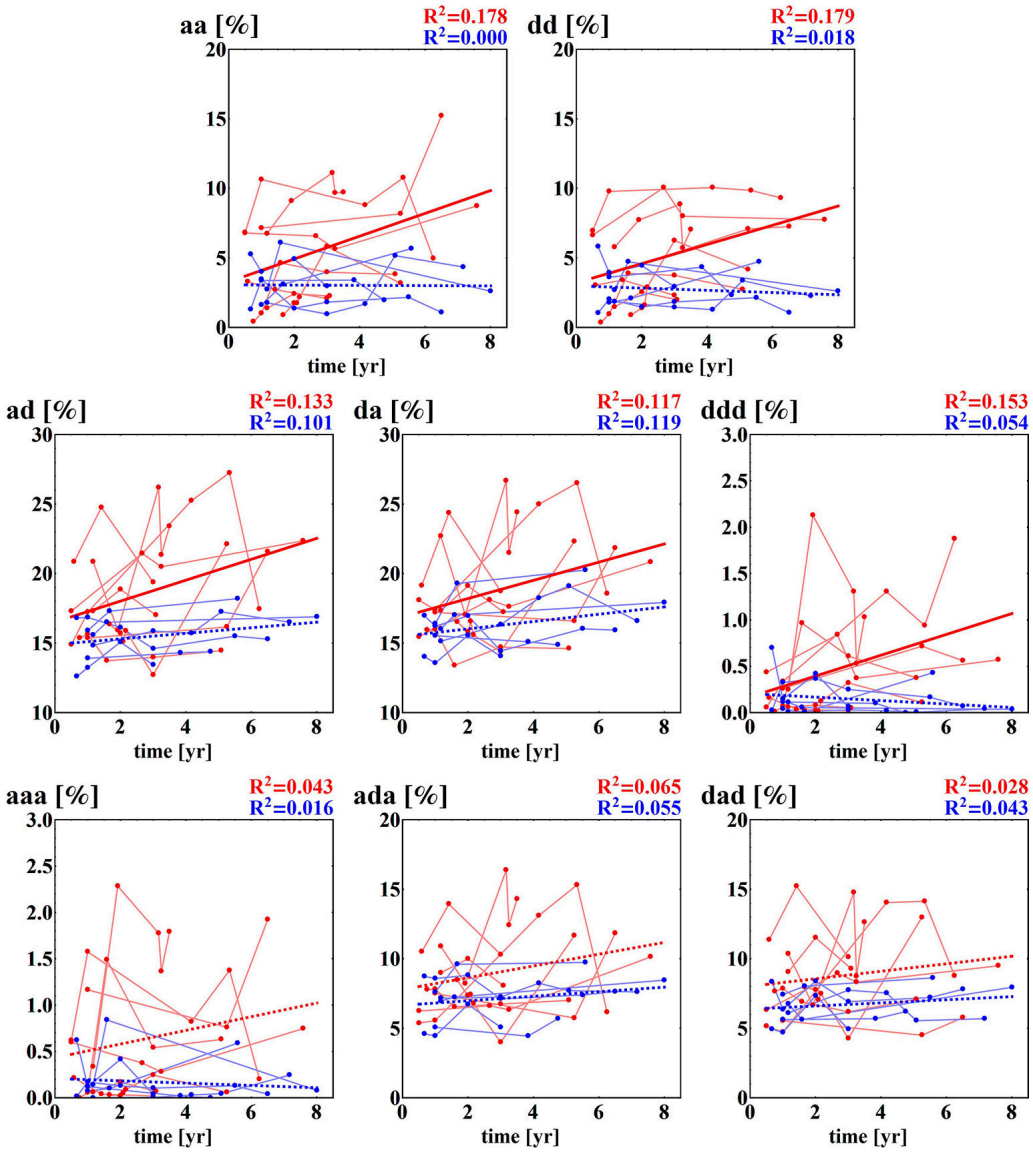
Time runs of fragmentation measures estimated for RR-increments obtained from the HTX groups. Blue marks and curves refer to the group of patients free of complications. Red notation is used for HTX patients with complications. The bold curves represent the best linear regression approximation to the group data. This line is dashed if the hypothesis of the zero regression coefficient cannot be rejected. For both lines, R^2^ correlation coefficients are shown. Points corresponding to the same patient are connected by faded lines.

Some of the fragmentation indices show a meaningful increase with time after surgery in the case of the NF group, while these indices stay constant in the case of the F group. This refers to events of two successive accelerations, two or three successive decelerations and two successive alternates: acceleration and deceleration in any order; see section (b) of Table 4. The relations of indices of particular patients over time are shown in Figure 5. The steepest increase is observed in the pattern of two accelerations. The properties described could provide hints at an explanation of the increases in the entropic measures discussed in the previous subsection for the NF group of HTX patients and stable values obtained for the patients in the F group.

## 4. Discussion

The healthy heart repeatedly and tirelessly alternates between accelerations and decelerations in response to the actual needs of the organism. It is believed that these changes follow the activity of the ANS (Task Force, 1996). The healthy human heart remains under the permanent influence of both branches of the ANS: the parasympathetic (considered to slow down HR) and the sympathetic (considered to speed up HR). Many measures estimating HRV have been proposed in order to quantify the regulatory function of the ANS (Task Force, 1996; Billman, 2011; Goldberger and Stein, 2015; Sassi et al., 2015; Laborde et al., 2017). Intensive healthy population studies have found a correlation between an increase in age and a decrease in many HRV indices, including indices such as RMSDD, pNN50 and HF, which describe the short-term variability (Reardon and Malik, 1996; Umetani et al., 1998; Pikkujämsä et al., 1999; Stein et al., 2009; Schumann et al., 2010; Makowiec et al., 2015b), which has been explained as being an impairment of heart adaptability with age. Also, reduced HRV has been associated with an adverse prognosis in patients with heart disease (Kleiger et al., 1987; Task Force, 1996; Bigger, 1997; DeJong and Randall, 2005; Thayler et al., 2010).

In the case of patients following HTX, any bicaval or biatrial technique interrupts neural conduction to the heart, though heart denervation is not complete as the intrinsic cardiac network, or ganglia of the transplanted heart, has the potential to function independently in the absence of central neuronal input (Armour, 2008; Karemaker, 2017). The HR becomes almost constant, which means that the HRV is very low (Ramaekers et al., 1996). Therefore it could be expected that any increase in HRV might be a manifestation of the regeneration of the autonomic regulation, possibly due to the process of reinnervation (Ramaekers et al., 1996; Cornelissen et al., 2012). However, the investigations presented here suggest that the increase in HRV indices results from the reconstruction in the myocardium due to complications in the patient after HTX, rather than from ANS regulation.

For our analysis, we selected long-term HTX patients whose progress after HTX was with complications. We decided to consider all the patients who suffered from fungal infection in their first year after the surgery (3 patients) and/or graft rejection (6 patients). All of them displayed hypertension (8 patients). Some were diabetics (7 patients), had chronic renal failure (4 patients), had suffered a stroke (3 patients) or had cytomegalovirus infection (3 patients); see Table 1 for details. The signals obtained from these patients were compared with signals recorded from HTX patients whose progress after the surgery was free of any complications although two of these patients, *F*_2_ and *F*_4_, displayed hypertension. Hypertension is noted in over 90 percent of HTX recipients (Lund et al., 2017), and is mainly connected with immunosuppressive drugs administration. In our patient groups, hypertension was treated according to cardiological standards with good results. No complications connected with hypertension were noted. To clarify whether hypertension was involved in the results obtained, we estimated separately the time development of HRV dynamical landscape indices of the two patients *F*_2_ and *F*_4_. We found that both patients perfectly displayed the *F* group properties, i.e., HRV indices were stable or going down with time. Therefore differences in HRV behavior in time, found between the two groups *F* and *NF*, encourage us to seek to understand HRV dynamic measures as the potential tools in the evaluation of patient prognosis.

Detailed studies of HTX patients have confirmed complete denervation within the first 1–6 months after HTX (Awad et al., 2016). There is evidence that sympathetic reinnervation progresses over time and increases even late after transplantation. It occurs that myocardial sympathetic reinnervation starts in basal parts of the anterior wall and subsequently progresses to distal parts of the myocardium. The parasympathetic nervous system reinnervates mainly atria, and to a much lesser extent the left ventricle (Grupper et al., 2017). Sympathetic reinnervation may occur without parasympathetic reinnervation, causing an unbalanced response to stimuli, but parasympathetic reinnervation seems to occur only in patients with sympathetic reinnervation. Parasympathetic reinnervation of the sinoatrial node is evaluated by respiratory sinus arrhythmia, which is connected mainly to vagal reinnervation (Crasset et al., 2001). An increase in HRV post-HTX might suggest vagal reinnervation, although some studies have failed to show any evidence of parasympathetic reinnervation (Grupper et al., 2017). In general, the reinnervation process appears in some but not all recipients, and it remains incomplete and regionally limited (Bravo et al., 2015; Grupper et al., 2017), and so is referred to as partial or patchy (Awad et al., 2016).

In contrast, among healthy people, especially at an elderly age, it has been observed that HRV may encompass not only autonomic modulation, but also variability from abnormal HR patterns. This phenomenon was referred to by Stein et al. (2002) as *erratic rhythm*, and consists of irregular sinus arrhythmia of non-respiratory origin (Stein et al., 2008; Nicolini et al., 2012; Costa et al., 2017). The erratic rhythm may have a confounding effect on age-related changes. Higher scores are observed, especially among short-term HRV indices (Makowiec et al., 2015a). The nature of erratic rhythms is unknown but, in general, the presence of the erratic rhythm impairs the prognostic power of HRV measures and age-related changes in HRV. Our investigations imply that the increase in short-term HRV indices observed in HTX patients may be related to the emergence of erratic rhythms. New methods are needed to distinguish ANS modulation from the erratic rhythm (Nicolini et al., 2012). The fragmentation indices introduced by Costa et al. (2017) are a proposition in this direction.

Erratic rhythm events are rather rare, and therefore long signals are required in order to obtain satisfactory statistics. We used signals consisting of 20,000 normal-to-normal RR-intervals, and we underline again that only sinus rhythm was considered. We decided to investigate nocturnal signals because of the supposed increase in vagal activity associated with sleep (Viola et al., 2004; Tobaldini et al., 2013; Chouchou and Desseilles, 2014), which slows down the heart rhythm, and in this way becomes a natural background against which the erratic rhythm can develop. An important factor was that none of the patients included in the study presented any sleep disorders caused by breathing. Moreover, the sleep recordings were substantially less perturbed by all kinds of artifacts than other parts of the Holter recording, and required minimal editing of the signals. Furthermore, as with normal sleep, there are prominent physiological rhythms associated with each approximately 90-min cycle of deep phase sleep with high vagal activity, followed by the REM phase with high sympathetic activity. This then switches between non-REM and REM, allowing insights into strong sympathetic and strong vagal activity and into the transition between these (Chouchou and Desseilles, 2014). The signals considered by us corresponded with approximately the first 4–6 h of sleep, hence two or three sleep cycles have been included.

Entropic measures are among the most frequently used methods of quantification of the signal complexity. They are based on the concept of the unpredictability of a state yielded by a system (Shannon, 1948). The entropy perfectly separates systems with the ideal order (the entropy is zero), when a system stays in the same state all the time, from completely random systems (entropy attains its maximal value), in which each state has an equal probability of occurring. In general, entropy provides an estimation of a given probability distribution of states (Hlavackova-Schindler et al., 2007). The dynamic aspects of an evolving system can be evaluated by relative quantities, such as the entropy rate *S*_*T*_ or self-entropy transfer *sTE* (Schreiber, 2000; Hlavackova-Schindler et al., 2007).

The visualization of HR dynamics presented in Figure 3 displays the crucial message of our analysis. It shows the mean diversity of the two successive states: actions of accelerations and decelerations, which remain stable over many years of uncomplicated progress after the surgery. The constant levels of all the HRV indices studied with the passing of time after the HTX for the patients in the F group, all together, support our main thesis that steady HRV serves well for the prognosis of the future state of a HTX patient.

The entropy estimates are driven by the action space, i.e., the way in which accelerations and decelerations are categorized. In the following, we have relied in the state space construction on the signal resolution. The action space and, consequently, the pattern classification are given with 8 ms accuracy. Such a resolution ensures that changes larger than 8 ms are discerned, though accelerations and decelerations of a size smaller than 8 ms cannot be evidenced. Therefore, the total of occurrences of the pairs of accelerations and decelerations *aa*, *ad*, *da*, and *dd* provided in Table 4 is far from 1. It is worth noting the similarity in the levels of the alternative patterns of *ad* and *da* between healthy coevals and HTX patients, together with the large discrepancy between these two groups in the case of the levels of the monotonic patterns *aa* and *dd*. This observation indicates possibly different mechanisms behind the dynamics of alterations in HR and behind the dynamics of speeding up or slowing down the HR. The alterations could be related to an accidental change in the velocity of impulse spreading the excitations in the myocardium tissue with the regular rhythm issued by the sinoatrial node. The minor occurrence of sequences of accelerations or decelerations in the HTX patients may be a manifestation of the effect of slow acceleration and deceleration ability after the HTX, verified, for example, by tests measuring the exercise capacity (Carvalho et al., 2009, 2013). Such a slow dynamics could be realized by patterns with “0,” namely patterns of *a*0*a* or *d*0*d* type are likely to be more common among the HTX patients than in the healthy group. Also, it is worth noting that the dynamical patterns studied, both monotonic and with alternations, are at significantly greater levels in the patients of the NF class than in patients of the F class; see Table 4. This could indicate a greater diversity of sources participating in the production of heartbeats.

The indices *H*_1_(Δ*RR*), RMSSD, and pNNx where *x* = 20 or 50, measuring the variability between two successive RR-intervals, provide estimates for a single RR-increment: its unpredictability, amplitude or frequency of occurrence, respectively. The results obtained are consistent with each other, independently of the differences in the measure concepts used in the construction of the short-term variability estimators. The levels of these indices found for HTX patients with complications, NF, are many times higher than for the F group patients, whose progress was free of complications. Moreover, all the indices display a tendency for an increase over time in patients in the NF group.

The entropy of a pair of successive RR-increments *H*_2_(Δ*RR*) and a triplet of successive RR-increments *H*_3_(Δ*RR*) provides HRV measures which are not accessible with standard HRV estimators. Similarly, the indices of fragmentation support standard HRV analysis. By considering metrics based on patterns classified due to the dynamical features they represent, we have achieved a comprehensive description of the dynamics of heart contractions. The entropic measures applied to the distributions of these patterns have provided us with a total measure of the fragmentation of a signal. They have confirmed the importance of the dynamics. The properties of *S*_*T*_ and *sTE* have proved the presence of non-stochastic drivers in this dynamics. Among these drivers, the erratic rhythm driver should be inspected. In particular, properties of *sTE* together with *H*_1_ have been found promising in discerning abnormalities in the rhythm as they appear to be the best discriminators between signals of the patients in the F and NF groups.

## 5. Conclusions

HRV is reduced immediately after HTX and may increase gradually with time, which is related to autonomic reinnervation. Beside the standard HRV indices, we have investigated features of segments with successive differences between heartbeats. For our analysis, we have selected long-term HTX patients who experienced complications after HTX or who were free of major complications. Differences in HRV found between these two groups encourage us to understand HRV dynamic measures as potential tools in the evaluation of patient prognosis.

The visualization of HR dynamics displays the crucial message of our analysis. It shows the mean diversity in patterns of the two successive states: actions of accelerations and decelerations, which remain stable over many years of uncomplicated progress after the surgery. The constant levels of all the HRV indices studied with the passing of time after the HTX in the patients in the F group, all together, support our main thesis that a steady HRV serves well in the prognosis of the future state of a HTX patient.

Our findings suggest that the increase in HRV indices observed is related to erratic rhythms resulting from remodeling of the cardiac tissue, including heterogeneous innervation, over-activated neurohormones, the aging process and immunosuppressive drugs taken chronically in long-term HTX patients. Therefore, we claim that short-term HRV indices can serve as a tool in the study of the genesis of non-respiratory sinus arrhythmia. Due to the regular observation of the heart rhythms of HTX patients, these data form a good starting point for this exploration.

## Author contributions

JW and MG: conceived and designed the study; JW: carried out the experimental protocol and collected the data; DM and ZS: established the dynamical landscape measures; DM and DW: performed data analysis, designed the visualization of the results; JW and DM: interpreted the findings; DM: drafted the manuscript; JW and DM: edited and revised the manuscript. All the authors read and approved the final manuscript.

### Conflict of interest statement

The authors declare that the research was conducted in the absence of any commercial or financial relationships that could be construed as a potential conflict of interest.
